# Assertiveness in the patients with thalassaemia major: A qualitative study

**DOI:** 10.1002/nop2.796

**Published:** 2021-02-23

**Authors:** Leila Valizadeh, Vahid Zamanzadeh, Akram Ghahramanian, Soghra Hasani Narenjbaghi

**Affiliations:** ^1^ Department of Pediatric Nursing School of Nursing and Midwifery Tabriz University of Medical Sciences Tabriz Iran; ^2^ Department of Medical‐Surgical Nursing School of Nursing and Midwifery Tabriz University of Medical Sciences Tabriz Iran

**Keywords:** assertiveness, nurse, qualitative research, thalassaemia major

## Abstract

**Aim:**

To explore the concept of assertiveness in the patients with thalassaemia major.

**Design:**

It was a qualitative study with a conventional content analysis design.

**Method:**

Data were collected using a semi‐structured interview. The research sample included 16 patients with thalassaemia major who were selected using purposeful sampling method from among such patients at educational and therapeutic centres. Then, they were analysed using MAXQDA10 software.

**Results:**

During the analysis of data for the concept of assertiveness, three sub‐concepts of "courage to self‐presentation," "demonstrating the abilities" and "attendance in groups" were developed.

## 1 INTRODUCTION

Assertiveness is one of the most important social skills (Motahari et al., 2019) that contributes to social‐cultural adaptation (Lee & Ciftci, 2014). It has been defined as the ability to express emotions, beliefs and explicit thoughts. Also, it could be considered as the ability to defend one's constructive and true behaviours and skills (Ahmadi et al., 2014) in a way that he/she could take action to obtain his/her rights without violating the rights of others (Speed et al., 2018). Individuals use assertiveness in the social interactions to create a sense of desirability (Baker & Jeske, 2015). So, programmes which are designed for assertiveness include constructing, maintaining and protecting the desired image of oneself. Assertiveness could lead to increase in the self‐efficacy (Motahari et al., 2019), social support (Rahimian Boogar et al., 2007), self‐esteem and interpersonal interactions (Lin et al., 2004; Manesh et al., 2015), improvement of the psychological status (Mc Cabe & Timmins, 2003) and decrease in the social anxiety (Lin et al., 2004; Manesh et al., 2015). Assertiveness could also be used for another purposes such as creating a positive self‐image (Malat et al., 2006). Patients with thalassaemia major have low self‐image (Jain et al., 2013) and dissatisfaction with their self‐image could affect such patients' education and sport activities (Ozturk et al., 2017). Assertiveness can be used to create a positive self‐image in these patients.

## 2 BACKGROUND

One of the most important factors in a healthy interpersonal relationship is the ability to be assertive (Motahari et al., 2019) that can lead to effective interpersonal relationship with others (Tavangar et al., 2014). People who could not be assertive, experience many problems including depression, resentment, disappointment, nervousness with anger, anxiety, poor social communication, physical complaints and family problems (Samouei et al., 2014). The results of Khazaie et al. (2014) showed that those who do not have assertive behaviours have lower self‐efficacy and self‐esteem, and higher social anxiety, shyness and aggression (Khazaie et al., 2014). People who fail to interact positively with others due to the lack of assertiveness or lack of interpersonal self‐efficacy will experience great anxiety when attending in a group and this may disrupt their social and occupational functioning (Tavangar et al., 2014).

Assertiveness could also affect health issues of people with chronic or serious diseases. It plays an important role in the life of such people, especially those belonging to the ethnic groups. Individuals from different ethnic groups may use assertiveness as a strategy to cope with ethnocentrism and prejudice in healthcare interactions (Jenerette & Dixon, 2010). Deprived individuals also use positive assertiveness as a strategy to improve their medical treatment (Malat et al., 2006). In patients with serious chronic diseases, assertiveness enhances positive ways to deal with the disease. According to the theory of self‐care management of sickle cell disease, assertiveness as a source of self‐care management has positive effects on health outcomes (Jenerette & Dixon, 2010). Since self‐care plays an essential role in the treatment of chronic diseases (Jaarsma et al., 2017), so, assertiveness can be an important strategy in such conditions.

One of the chronic and genetic blood diseases is thalassaemia (Elzaree et al., 2018). In this disease which is more prevalent in the Mediterranean countries (Behnam Vashani et al., 2015), the patient needs repeated blood transfusion and use of iron chelating drugs to survive (Ishfaq et al., 2018). In Iran, there are about 25000 people with this disease (Razzazan et al., 2014). These patients have several physical problems including severe chronic anaemia, hepatosplenomegaly, bone changes especially in face and head (Zarea et al., 2012), facial pigmentation, growth failure (Elzaree et al., 2018) and pubertal delay or failure (Ozturk et al., 2017). In addition to the physical problems, patients also suffer from the psychosocial problems such as depression, anxiety, anger, limitation in the social communication (Ozturk et al., 2017), social isolation, aggression, decrease in self‐esteem and being different (Ishfaq et al., 2018).

Review of the literature has shown that assertiveness is helpful in the chronic patients' adjustment. The role of assertiveness in the self‐management of pain in patients with sickle cell disease has been shown in some of the studies (Jenerette & Dixon, 2010). Also, Khayyam Nekouei et al. (2010) used the cognitive‐behavioural training, a part of which was training the assertiveness skill, to improve the life quality of the cardiac patients. As a result, the cardiac patients' life quality improved satisfactorily (Nekouei et al., 2010).

As it was previously mentioned, assertiveness is very important in the interpersonal communication and social interactions. Since thalassaemia patients have many problems with social communication due to the facial changes and disease stigma (Jain et al., 2013), assertiveness can be an important strategy to help these patients to communicate. Due to this fact, the researchers decided to conduct a qualitative study to answer the question on "how do the patients with thalassaemia become assertive?" Qualitative research is a way of discovering the depth, richness and inherent complexity of a phenomenon and reveals its various aspects (Speziale et al., 2011).

## 3 METHODS

### 3.1 Design

Due to the purpose of the study and to explore the concept of assertiveness in patients with thalassaemia major, a qualitative study using the conventional content analysis technique was used. Using this technique, a valid interpretation is provided from the data, considering their context with the purpose of creating knowledge, acquiring new insights, expressing the facts and developing a practical guide (Elo & Kyngäs, 2008).

### 3.2 Setting and participants

The researcher explained the purpose of the study to the patients with thalassaemia major who referred to thalassaemia major ward of Bouali Sina(Sari) and Shahid Ghazi (Tabriz) educational medical centres in Iran for blood transfusion. Sixteen patients who announced their willingness to participate in the study were selected as the research sample, taking into account their diversity in terms of socio‐economic status, education, occupation, gender, age, marital status and occupation. They were selected using purposeful sampling method and the informed written consent was obtained from them by the researcher (the corresponding author). Demographic characteristics of the participants are presented in Table 1.

**TABLE 1:** The demographic characteristics of the participants

sex	age	education	marital status	Job
Male	18	High school	Single	unemployed
Female	17	High school	Single	unemployed
Male	26	BA	Single	Web designing
Female	35	Diploma	Married	The board of directors of NGO
Male	34	Primary school	Married	worker
Female	37	BA	Married	Government employee
Male	37	Guidance School	Married	Freelance job
Female	21	Diploma	Married	housewife
Female	36	Diploma	Divorced	Freelance job
Female	27	Diploma	Single	Freelance job
Male	30	BA	Single	Private centre employee
Female	38	BA	Single	Private centre employee
Female	27	MA	Married	student
Male	22	Diploma	Single	Freelance job
Female	18	High school	Single	student
Female	22	BA	Single	unemployed

### 3.3 Data collection

Interviews were conducted by the corresponding author who has lots of experiences in attending the qualitative interview workshops and qualitative research classrooms during her Ph.D. programme. To collect the data, semi‐structured interview with open‐ended and exploratory questions, as well as field notes, were used until the theoretical saturation was achieved, that is until there was no new code or data. Actually, the data were saturated after 13th interview and 3 more interviews were conducted to ensure that no new data could be obtained. Two examples of the open‐ended questions were as follows:

How does your illness affect your communication with others?” and “How do you explain your illness to others?

Location of the face‐to‐face interview was determined by the participants. They were interviewed individually and each interview took between 30–65 min in a private room. All the interviews were audio‐recorded and there was no repeated interview. Also, no participant refused to continue the study.

### 3.4 Data analysis

Data were analysed using the conventional content analysis technique. Here, the researchers did not use the predetermined categories, instead they allowed the categories and names of the categories to be continuously generated from data (Hsieh & Shannon, 2005). After specifying the unit of analysis, assigning a code to each meaning unit and putting the codes with similar meaning into the subcategories and categories, main themes of the meaning units were identified (Elo & Kyngäs, 2008). In this study, the conventional content analysis was conducted using Wildemuth method. According to Wildemuth's proposed stages, at the data preparation stage, all the interviews are transcribed, and then, at the next stage, the unit of analysis is specified (Wildemuth, 2017). In this study, interviews with thalassaemia major patients were considered as the unit of analysis. These interviews were listened several times immediately after they were conducted, and then, they were transcribed along with non‐verbal communication of the participants. In order to become immersed in the interview texts and gain an overall sense of their meaning, they were read and reviewed several times like a story. Then, the words of the interviews, which suggested the concept of assertiveness of patients with thalassaemia major, were identified; some codes were assigned to them and the initial coding scheme was obtained. During the coding, it was attempted to extract and codify the explicit and hidden meanings of the meaning units. Then, the codes with similar meaning were put into a subcategory and then into a category. The continuous comparison method was used to gain an insight and understanding of the differences between the categories. Data were analysed using MAXQDA 10 software.

### 3.5 Ethics

This study was approved by the Ethics Committee of Tabriz University of Medical Sciences under the code of ethics IR.TBZMED.REC.1397.616. After giving a brief explanation about the research, a written informed consent was completed and signed by the research participants. Then, the researcher began to interview the patients who were willing to participate in the study. In order to record the participants' statements, their permission was taken and they were assured that their statements would be kept confidential.

### 3.6 Rigour

Criteria used by Lincoln and Guba were used in this study to ensure the accuracy and reliability of data (Lincoln & Guba, 1985). In order to validate the data, the extracted original codes were returned to the participants, so that correctness of the researchers' interpretation of what they said could be confirmed. It was also attempted to validate the data by considering maximum variation in the sampling (diversity of the research participants in terms of socio‐economic status, education, occupation, gender, age, marital status and occupation), adequate participation and close interaction with the participants. Methods of determining the dependability in this study included prolonged engagement of the researcher with the data, coworker review and participant review. Transferability of the findings was established through presenting the quotes of the participants in the same way as they were stated, as well as providing demographic characteristics of the participants in detail, so that the reader could decide on the use of the results of the study. And the conformability of the data was ensured through reviewing by the external examiners, that is parts of the interview transcript along with the relevant codes and emerged categories were examined by four examiners familiar with the qualitative research and one psychologist. To construct the audit trail of the research, the researcher accurately recorded and reported the research process and procedures, so that others could follow it with ease.

## 4 RESULTS

Through analysing the data, three sub‐concepts and one main concept emerged (Table 2). One of the most common experiences of patients with thalassaemia major is concealment of the disease in order to not getting the disease stigma, getting the desired job and getting married. The disease concealment has physical, psychological and social consequences. Therefore, assertiveness is an important concept in maintaining health in these patients, and it includes the three sub‐concepts: «courage to self‐presentation», «demonstrating the abilities» and «attendance in groups» (Table 2), which would be investigated in what follows, respectively.

**TABLE 2:** Codes, preliminary and main concept of the assertiveness

Main concept	Preliminary concept	Code
		Talking about one's disease with the coworkers
		Talking about one's disease with the friends
		Talking about one's disease for the public
	The courage to the self‐presentation	Talking about one's disease and its complications with the suitor
		not concealing one's disease
Assertiveness	Demonstrating the abilities	Trying to demonstrate one's abilities to the others
		Trying to prove oneself in the workplace
		Encouraging the other patients to attend in the association
	Attendance in the groups	Form the thalassaemia association
		Member of the healthy individuals' indoor football team
		Member of the bureau's mountaineering group

Since assertiveness is a way of expressing oneself to realize his/her rights without violating the rights of others, so, courage to self‐presentation, to show one's abilities, and to attend in a group is an example of assertiveness in patients with thalassaemia major. Considering the concept of «courage to self‐presentation», person with thalassaemia wants to establish a social relationship, free from fear and anxiety, regardless of his/her physical problems. So he/she introduces himself/herself as a person with such illness among the colleagues, friends and suitor, and he/she is not afraid of revealing his/her illness.

Each person can use his/her abilities to achieve his/her goals. In cases where others consider the person as incapable, he/she can achieve his/her inalienable right to occupy a professional and social position by demonstrating his/her abilities. Considering the concept of «demonstrating the abilities», person with thalassaemia tries to show his/her abilities and prove himself/herself by hard‐working in the workplace.

One of the components of responsibility in the emotional intelligence is self‐presentations of a member with a sense of effective and constructive cooperation in the group. Membership of patients with thalassaemia in sport teams, and non‐governmental organizations and encouraging others to participate in the activities of the related organizations is a sign of their responsibility towards the teams of which they are members of and also towards their peers. In these patients, assertiveness is seen as a symbol of «responsibility».

### 4.1 The courage to self‐presentation

Failure in introducing himself/herself as a thalassaemia patient has adverse consequences for these patients. For example, the employers' unwillingness to employ thalassaemia patients due to their repeated absences for the treatment such as periodic blood transfusions and intravenous injections of iron chelating drugs results in the concealment of disease by the patients. So, the patient cannot take blood transfusions within the prescribed time, and following it, the complications due to anaemia leads in the physical disability and other problems for the patient. In this regard, one participant said:

the peers, especially our boys, go to work somewhere, if they say they have thalassaemia, undoubtedly the employer won't accept them. If they conceal it, they will face problems that harm them. (P: 4)

These patients have certain right to have healthcare facilities and necessary treatment. The thalassaemia patient should have the courage to present himself as such a patient in the workplace. In this regard, one participant said:

In my workplace, there are 80 people who know I am sick. I told them myself… I explain them I need blood transfusion and I take drugs. (P: 12)

Another case of concealment of disease which also brings some problems for these patients is marriage with healthy people. When these patients conceal their disease at the time of getting married, they have to either ignore their blood transfusions or not to do it regularly. In this regard, a participant commented about her friend's concealment of the disease from her spouse:

Our peers do not succeed in marrying healthy people. They do not tell their husband's families that they have this disease. Even one of our peers died because of this. (P: 13)

Marriage is the right of any person which should be done honestly in order to prevent infringement of the right of the other side. These patients also have the right to marry a healthy person or a peer. One participant commented about her disease and the right to marry honestly:

when I got married I explained him everything and even I said I have a blood problem; we may not have a baby or maybe I could not give birth to a healthy baby. (P: 9)

Patients who do not hide their illness experience fewer psychological and social problems. In this regard, one of the participants said:

If one day I do not go to work, my friends will call me and ask why I didn't come to work. We are very close friends. My mental state is much better than those who hide their illness. Generally, my status is better than them. (P:12)

### 4.2 Demonstrating the abilities

Assertiveness is a condition of interaction and an integral part of social life. People seek social approval in social communication. This issue is highlighted in the case of thalassaemia patients because of their physical appearance. They want to demonstrate their abilities in their communication to achieve social desirability. In this regard, one participant stated:

I am ready to do everything to make healthy people realize that I have thalassaemia, however, I have no problem in terms of thinking, physical or behavioral abilities. (P: 11)

In addition, one of the problems of these patients is the belief of others about their abilities. Because they have developmental delay and delayed puberty, people consider them weak and powerless. Also, because of their need to regular blood transfusions and time‐consuming treatment of iron‐depleting drugs, people, especially employers, could not recognize them as a useful workforce. Such beliefs exacerbate their psychological problems such as social isolation and depression. These patients know that they could get rid of such problems if they can get a job and maintain it. So, they try to show their abilities to others and to prove themselves in the workplace. In this regard, one participant commented:

When the employer realizes that you are sick, you try to work in such a way to change his opinion and viewpoint about yourself, and proving happens right now. (P: 14)

### 4.3 Attendance in groups

Patients with thalassaemia benefit from being in groups to overcome their problems. Due to their physical condition and stigma, these patients usually isolate themselves socially, which leads in lots of psychological damages including depression. Attendance in groups leads to a reduction in psychological problems including low self‐esteem of these patients. By accepting responsibility and participating in groups, they realize their values and as a result, their self‐esteem improves. Another benefit of attendance in groups is increase in the interaction with others. In this regard, one of the participants stated:

When I'm in the association, I work with patients and those who are there, I'm in a good mood. I feel I am more valued. (P: 4)

An effective and constructive attendance in social groups leads in self‐satisfaction. Following the self‐satisfaction, thalassaemia patients also try to achieve a personal satisfaction and to attain a desirable social status in their peer and healthy individual social groups through their effective and constructive presence.

Attendance in groups is an essential part of accepting responsibility and social cooperation. It is a form of conscious, voluntary and purposeful involvement of individuals in the process and social affairs to achieve the group goals or development, facilitating the group affairs and exploiting their results. In this regard, a participant who was the member of a non‐governmental institution said:

Our city had a youth institute and we were its members, all members were thalassaemia patients and its head had thalassaemia, too. There was a non‐governmental institution that worked for thalassaemia; we went there to help them. (P: 6)

Another participant who was a member of a sport team commented on his role in the team:

I go to the gym where I play in a healthy group. We have a good indoor football team. We work well with each other for the success of the team. (P: 11)

## 5 DISCUSSION

Findings of this study showed that assertiveness is an important concept in patients with thalassaemia major. In our study, the patients considered introducing themselves as the thalassaemia patients who could work according to their physical condition and treatment programme and could get marry or educate, as a part of the concept of assertiveness. They commented that when interacting with individuals such as employer, coworkers, classmates and suitor, assertiveness is an important strategy. The results of Malat et al. (2006) have also shown that in the situations where one interacts with other powerful individuals, positive assertiveness is an important characteristic (Malat et al., 2006).

Patients with thalassaemia major use concealment strategy to escape the disease stigma due to the misconceptions in the society. Physical exhaustion, psychological distress such as anxiety and fear of disclosure of disease, depression and lack of access to others' support are the consequences of using concealment strategy (Pouraboli et al., 2014). Introducing himself/herself as a patient with thalassaemia is the opposite of concealment strategy. In the present study, the participants who did not conceal their disease from others and showed assertiveness through introducing themselves as patients with thalassaemia major acknowledged to have high self‐esteem, and also not to have mental problems such as anxiety.

The results of several studies were in line with the results of our study. For example, in a study, there has been a positive relationship between assertiveness and self‐esteem and a strong and inverse relationship between assertiveness and anxiety of nursing and midwifery students (Shrestha, 2019). The inverse relationship between assertiveness and anxiety has also been reported among nutritionists (Paterson et al., 2002).

The results of a study based on the hypothesis on the relationship between social anxiety and emergence of less assertive behaviours showed that such behaviours have been associated with higher social anxiety (Weber et al., 2004).

In this study, patients with thalassaemia major have considered assertiveness as an important strategy to prove themselves in the workplace and maintain their occupational status. Despite increase in the public awareness about thalassaemia, there are still many previous attitudes towards these patients. The others' inappropriate reactions could affect such patients' ability in the workplace. Therefore, in the work environment, these patients try to prove that they are not different from other coworkers through demonstrating their physical and thinking abilities to their employer and the coworkers. Their efforts to demonstrate their ability through assertiveness play an important role in maintaining their job. Emotional intelligence and its components including assertiveness have an influential role on performance in the workplace and also on evaluation of one's performance by himself, coworkers or managers (Golparvar & Khaksar, 2010).

Therefore, findings of our study on showing one's abilities as an assertive behaviour are in line with the theories on the effectiveness and importance of emotional intelligence and its components on the behaviours in the work and social environments (Day & Carroll, 2004; Dulewicz & Higgs, 2000).

One of the examples of assertiveness mentioned by the participants was attendance in groups (including thalassaemia groups and healthy individuals' sport teams). Mental and psychological problems caused by the disease and inappropriate reaction of others could cause the patient with thalassaemia major to stay away from the society. The results of Ishfaq et al. (2018) showed that thalassaemia major leads to socio‐psychological problems including social isolation. 54% of the participants in this study did not participate in sport activities (Ishfaq et al., 2018). Patients with thalassaemia major are assertive through joining into peer groups and sport teams and having a collaborative and constructive effect in the groups as an effective member. The participants' comments about attendance in groups as a characteristic of assertiveness along with the ability to express themselves in the form of a cooperative, effective and constructive member of the group are consistent with the component of responsibility in the Bar‐On emotional intelligence scale (Ahmadi et al., 2014).

### 5.1 Limitations and strong points of the study

The use of a qualitative study to understand the concept of assertiveness in patients with thalassaemia major is a strong point and lack of ethnic diversity among the participants is a limitation of the present study. We suggest participants with more ethnic diversity to be selected in the future studies.

## 6 CONCLUSION

In the present study, assertiveness was developed with the three sub‐concepts of «courage to self‐presentation», «demonstrating the abilities» and «attendance in groups». Assertiveness as a coping strategy could help patients to cope with the psychological burden of thalassaemia.

Stigma can affect patients with thalassaemia's mental health, physical function, daily roles and social function. Assertiveness can be considered as a strategy to overcome the stigma of these patients and it could lead to improvement of their mental, physical and social health.

## 7 RELEVANCE TO THE CLINICAL PRACTICE

Lack of assertiveness is an interpersonal problem that can affect social, occupational, family status and social interactions (Maddahi et al., 2011). Assertiveness has a direct relationship with academic achievement, and it reduces anxiety (Mohebi et al., 2012), social anxiety (Ahmadi et al., 2017) and depression (Rezayat & Nayeri, 2014). Through assertiveness, one can easily express his/her needs and beliefs without anxiety, thus making interpersonal relationship more effective by properly expressing thoughts and feelings. Nurses and healthcare workers need to be aware of the role of assertiveness and create training assertiveness programmes for thalassaemia patients to help them to overcome their problems.

## CONFLICT OF INTEREST

The authors have declared no conflict of interest.

## Data Availability

The data that support the findings of this study are available from the corresponding author (SHN) upon reasonable request.
